# Likely questionnaire-diagnosed food allergy in 78, 890 adults from the northern Netherlands

**DOI:** 10.1371/journal.pone.0231818

**Published:** 2020-05-13

**Authors:** Cornelia Doriene Westerlaken-van Ginkel, Judith M. Vonk, Bertine M. J. Flokstra- de Blok, Aline B. Sprikkelman, Gerard H. Koppelman, Anthony E. J. Dubois

**Affiliations:** 1 Department of Paediatric Pulmonology and Paediatric Allergy, University of Groningen, University Medical Center Groningen, Groningen, The Netherlands; 2 University of Groningen, University Medical Center Groningen, GRIAC Research Institute, Groningen, The Netherlands; 3 Department of Epidemiology, University of Groningen, University Medical Center Groningen, Groningen, The Netherlands; 4 General Practitioners’ Research Institute, Groningen, The Netherlands; University of Tasmania, AUSTRALIA

## Abstract

**Background:**

It is challenging to define likely food allergy (FA) in large populations which limited the number of large studies regarding risk factors for FA.

**Objective:**

We studied the prevalence and characteristics of self-reported FA (s-rFA) in the large, population-based Dutch Lifelines cohort and identified associated risk factors.

**Methods:**

Likely food allergic cases (*LikelyFA*) were classified based on questionnaire reported characteristics consistent with FA. Subjects with atypical characteristics were classified as *Indeterminate*. We investigated 13 potential risk factors for *LikelyFA* such as birth mode and living on a farm and addressed health-related quality of life (H-RQOL).

**Results:**

Of the 78, 890 subjects, 12.1% had s-rFA of which 4.0% and 8.1% were classified as *LikelyFA* and *Indeterminate*, respectively. Younger age, female sex, asthma, eczema and nasal allergy increased the risk of *LikelyFA* (p-value range <1.00*10^−250^–1.29*10^−7^). Living in a small city/large village or suburb during childhood was associated with a higher risk of *LikelyFA* than living on a farm (p-value = 7.81*10^−4^ and p = 4.84*10^−4^, respectively). Subjects classified as *Indeterminate* more often reported depression and burn-out compared to those without FA *(*p-value = 1.46*10^−4^ and p = 8.39*10^−13^, respectively). No association was found with ethnicity, (duration of) breastfeeding, birth mode and reported eating disorder. Mental and physical component scores measuring H-RQOL were lower in both those classified as *LikelyFA* and *Indeterminate* compared to those without FA.

**Conclusion:**

The prevalence of s-rFA among adults is considerable and one-third reports characteristics consistent with *LikelyFA*. Living on a farm decreased the risk of *LikelyFA*. The association of poorer H-RQOL as well as depression and burn-out with questionable self-perceived FA is striking and a priority for future study.

## Introduction

Food allergy (FA) is defined as an adverse health effect arising from a specific immune response that occurs reproducibly on exposure to a given food [1]. These immediate, IgE mediated reactions to food have a high impact, both socially and financially [2,3]. Previous research indicated that self-reported FA (s-rFA) was associated with psychiatric disorders such as depression and internalization of problems [4,5]. Despite the major impact it has on patients and their families, the pathogenesis of FA remains poorly understood and so far, there is little knowledge regarding the characteristics of subjects with s-rFA in the general population.

In 1994, the prevalence of FA and food intolerances was studied by a questionnaire in a random sample of 1 483 Dutch adults. Approximately 12.4% answered ‘yes’ to “Do you have allergic or intolerance reactions after eating or drinking specific foods; or are there any foods you do not use anymore because they give you trouble?” [6]. In only 12/73 subjects (16.4%) with s-rFA or food intolerance, could this be confirmed by either a positive double-blind placebo-controlled food challenge (DBPCFC n = 9) or a prolonged glucose tolerance test (n = 3) [6]. The latter was performed in subjects suspected of glucose intolerance. Moreover, the foods listed are more likely to be involved in food intolerance based on a non-allergic adverse reaction and the DBPCFCs protocol was highly different from current standards [7]. In a more recent study, 25% of 3 864 Dutch adults reported adverse reactions to foods [8]. A meta-analysis described the prevalence of FA in European adults using the following definitions; s-rFA, s-rFA accompanied by positive sIgE and challenge proven FA [9]. The prevalence according to these definitions was 5.1%, 2.2% and 0.1–3.2%, respectively. This shows that the prevalence of FA is considerable, but highly dependent on the used definition [10].

In a telephone survey among 5 300 households in the US, Sicherer *et al*. documented that 18/93(19.4%) adults with s-rFA had no convincing reaction based on reported symptoms and timing of onset of symptoms [11]. In a two-staged questionnaire among 1 583 adults from Central Brazil, Silva *et al*. found that 89/104(85.6%) subjects with s-rFA were not considered to have FA based on the reported food, symptoms, timing of onset and reproducibility of symptoms, and effect of food exclusion [12]. With a false positive rate between 19.4% and 85.6%, these studies show that s-rFA overestimates the prevalence of FA.

This study, based on the population-based cohort Lifelines [13], aimed to describe the prevalence of likely as well as questionable, self-perceived FA among Dutch adults and to identify risk factors for both conditions. We were interested in the association with age, gender, other atopic diseases, mode of delivery, breastfeeding and early farm exposure. Cases with likely FA were defined as those who reported foods, symptoms and characteristics consistent with FA. In this definition, we aimed to optimize the specificity for FA. Controls were those who reported that they did not have FA. The remainder, classified as *‘Indeterminate’*, reported FA but with foods, symptoms and/or characteristics other than those consistent with FA. By studying the association of mental disorders and health-related quality of life (H-RQOL) with cases, controls and this ‘*Indeterminate*’ group, we aimed to further characterize these populations. Of special interest in this regard is the *Indeterminate* group with questionable, self-perceived FA, about which very little is currently known, despite its substantial prevalence.

## Methods

### Lifelines

Lifelines is a multi-disciplinary prospective population-based cohort study examining, in a unique three-generation design, the health and health-related behaviors of 167,729 persons living in the northern Netherlands. It employs a broad range of investigative procedures in assessing the biomedical, socio-demographic, behavioral, physical and psychological factors which contribute to the health and disease of the general population, with a special focus on multi-morbidity and complex genetics [13,14]. This cohort is broadly representative of socioeconomic characteristics, lifestyle, diseases and general health of the population in the northern Netherlands [15]. Recruitment of participants was performed between 2006 and 2013. From 2014 onwards, subjects were invited to complete a second examination, including the “Food Allergy Questionnaire” (FAQ). We included adults who completed this before 01-01-2017. Children received the FAQ after this date and were therefore not included. The LifeLines cohort study was approved by the Medical Ethics Committee of the University Medical Center Groningen, Groningen, The Netherlands (2007/152). All subjects gave written informed consent.

### Classification of subjects

Subjects were classified as ‘not having FA’ (*NoFA*), ‘likely to have FA’ (*LikelyFA*) or *Indeterminate*. Our aim was to maximize the specificity of the FAQ for the classification of *likelyFA* since we wanted to distinguish immediate allergic reactions to food from other (non-)allergic food hypersensitivities or intolerances. Subjects were classified as ‘*NoFA*’ if they answered ‘I do not have food allergy’ to Question 1 (Which of the following food-items cause an allergic reaction?).

**Fig 1:**
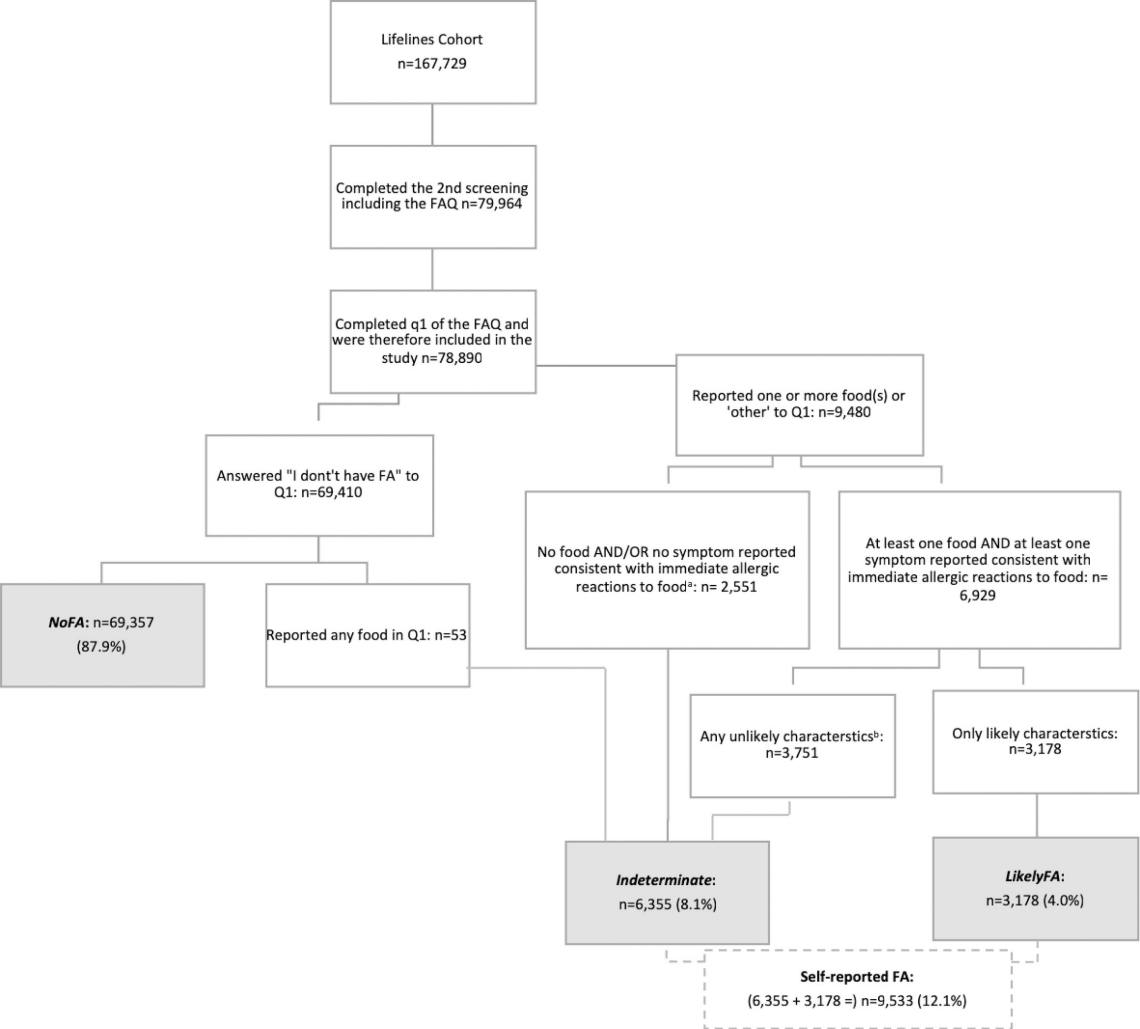
Flowchart of the study population and food allergy classification. FAQ = Food Allergy Questionnaire. Q1 = question 1 (Which of the following food-items cause an allergic reaction?). NoFA = no food allergy, LikelyFA = likely food allergy. As indicated, only subjects who reported at least one food and at least one symptom consistent with immediate allergic reactions to food and who reported only likely characteristics of food allergy were classified as *LikelyFA*. All other cases who reported any food in Q1 were classified as *Indeterminate*. ^a^ n = 372 only reported a food which caused an allergic reaction, which is not described in S1 Table since it was reported in less than 1:1000 of patients with s-rFA. Furthermore, n = 312 only reported something other than a food such as ‘I don’t know’,’ currently under investigation’ or ‘not applicable’. ^b^ These unlikely characteristics are symptoms appearing after a day or more, following at least a normal portion or more, symptoms persisting for >1 week, a diagnosis by an alternative practitioner without a diagnosis by any clinician and a negative double-blind, placebo-controlled food challenge, see Table 1.

Subjects were classified as ‘*LikelyFA’* if they reported:

■ at least one food (e.g. apple, peanut, egg, milk) consistent with immediate allergic reactions to food AND
■ at least one symptom (e.g. diarrhea, urticaria, wheezing) consistent with immediate allergic reactions to food AND
■ other characteristics of FA consistent with immediate allergic reactions to food (listed in Table 1 and Fig 1).

**Table 1:** Food allergy classification in the Lifelines study population. Variables highlighted are consistent with immediate allergic reactions to food. Variables not highlighted were classified as not consistent with immediate allergic reactions to food.

Translated question (English)	1. Which of the following food-items cause an allergic reaction?	Other(s) namely …..^*c*^	2. Which symptoms occur after eating or drinking the food item you are allergic to?	Other(s) namely ….. ^*c*^	3. Who diagnosed the food allergy?	4. Do you have an adrenalin auto-injector /Epipen /Anapen/ Jext ?	5a. Were you tested in a 2-day double blind oral food challenge?	5b. Did this test show that you are allergic for at least one food?	6a. Which food item triggers the most severe allergic reaction?	Other(s) namely …. ^*c*^	6b. How quickly do these symptoms appear?	6c. Which amount causes these symptoms?	6d. How long do these symptoms persist?
**Original question (Dutch)**	1. Voor welke van deze voedingsmiddelen bent u vermoedelijk allergisch?	Anders namelijk …..^*c*^	2. Welke klachten ontstaan na het eten of drinken van voedingsmiddelen waar u allergisch voor bent?	Anders namelijk…..^*c*^	3. Door wie is de voedselallergie vastgesteld?	4. Heeft u een adrenaline auto-injector / Epipen/ Anapen/ Jext?	5a. Heeft u een tweedaagse (dubbelblinde) voedsel provocatietest ondergaan?	5b. Kwam uit deze test dat u allergisch bent voor tenminste een voedingsmiddel?	6a. Van welk voedingsmiddel krijgt u de **heftigste** allergische reactie? *(Slechts één antwoord mogelijk)*	Anders namelijk….^*c*^	6b. Hoe snel ontstaan deze klachten?	6c. Van welke hoeveelheid ontstaan de klachten?	6d. Hoe lang houden de klachten aan?
**Type of answers**	*Multiple answers possible*	*Written answers*^*c*^	*Multiple answers possible*	*Written answers*^*c*^	*Multiple answers possible*	*Only one answer possible*	*Only one answer possible*	*Only one answer possible*	*Only one answer possible*	*Written answers*^*c*^	*Only one answer possible*	*Only one answer possible*	*Only one answer possible*
**Reference**	n^T^, (% of total (78, 890)) *[n*^*L*^ *(% of n*^*T*^ *) classified as LikelyFA] (when applicable)]*	n^T^, (% of total (78, 890)) *[n*^*L*^ *(% of n*^*T*^ *) classified as LikelyFA]*	n (% of subjects reporting any food n = 9 480)	n (% of subjects reporting any food n = 9 480)	n (% of subjects reporting any food n = 9 480)	n (% of subjects reporting any food n = 9 480)	n (% of subjects reporting any food n = 9 480)	n (% of 9a: Yes = 189)	n (% of subjects reporting any food n = 9 480)	n (% of subjects reporting any food n = 9 480)	n (% of subjects reporting any food n = 9 480)	n (% of subjects reporting any food n = 9 480)	n (% of subjects reporting any food n = 9 480)
	I don’t have food allergy^a^ n = 69 410 (88.0)	Kiwi n = 776 (1.0) *n = 480 (61*.*9)]*	Abdominal cramps n = 2935 (31.0)	Painful mouth/tongue n = 157 (1.7)	I did it myself n = 6620 (69.8)	No n = 8764 (92.4)	No n = 8817 (93.0)	Yes n = 144 (76.2)	Cow’s milk n = 1162 (12.3)	Kiwi n = 518 (54.6)	Minutes–one hour n = 3886 (41.0)	Crumbs–few bites/sips n = 2495 (26.3)	Several hours n = 2944 (31.1)
	Any food such as below or other n = 9 480 (13.1) *[n = 3 178 (33*.*5)]*	Strawberry n = 236 (0.3) *[n = 92 (39*.*0)]*	Itch in mouth/ear/throat n = 2742 (28.9)	Red/swollen eyes n = 47 (0.5)	Family doctor n = 1456 (15.4)	Yes n = 150 (1.6)	Yes n = 189 (2.0)	No n = 31 (16.4)	Apple n = 1026 (10.8)	Strawberry n = 125 (13.2)	Immediately (seconds) n = 2142 (22.6) ^g^	Traces (invisible) n = 518 (5.5)	<1 hour n = 2725 (28.7)
	Apple n = 1 972 (2.5) *[n = 1112(56*.*4]*	Cherry n = 170 (0.2) *[n = 119 (70*.*0)]*	Itch on tongue and/or lips n = 1853 (19.5)	Sneezing n = 47 (0.5)	Dermatologist n = 621 (6.6)	*I don’t know n = 498 (5*.*3)*	*I don’t know n = 406 (4*.*3)*	*I don’t know n = 14 (7*.*4)*	Shellfish n = 671 (7.1)	Cherry n = 62 (0.7)	After a few hours n = 1773 (18.7)	*I don’t know n = 1825 (19*.*3)*	Several days n = 1252 (13.2)
	Cow’s milk	Pear n = 162 (0.2) [*n = 123 (75*.*9)]*	Diarrhea n = 1835 (19.4)	Strange feeling/painful throat n = 40 (0.4)	Allergist n = 457 (4.8)	*Missing n = 68 (0*.*7)*	*Missing n = 68 (0*.*7)*		Wheat n = 631 (6.7)	Carrots (peel) n = 46 (0.5)	*Missing n = 194 (2*.*0)*	*Missing n = 211 (2*.*2)*	Day n = 1196 (12.6)
	n = 1 877 (2.4) *[n = 414 (22*.*1)]*												
	Hazelnut n = 1 634 (2.1) *[n = 979 (59*.*9)]*	Peach n = 160 (0.2) *[n = 112 (70*.*0)]*	Tightness of throat n = 1713 (18.1)	Swollen face n = 24 (0.3)	Dietician n = 214 (2.3)				Hazelnut n = 608 (6.4)	Peach n = 43 (0.5)	*I don’t know n = 685 (7*.*2)*	Normal portion or more n = 4431 (46.7)	*Missing n = 203 (2*.*1)*
	Walnut	Fruit n = 141 (0.2) *[n = 96 (68*.*1)]*	Nausea n = 1654 (17.4)	Swollen throat n = 23 (0.2)	Pediatrician n = 102 (1.1)				Walnut n = 483 (5.1)	Pear n = 35 (0.4)	After a day or more n = 800 (8.4)		*I don’t know n = 875 (9*.*2)*
	n = 1 433 (1.8)												
	*[n = 839 (58*.*5)]*												
	Wheat^b^	Banana n = 113 (0.1) *[n = 57 (50*.*4)]*	Swelling of tongue and/or lips n = 1484 (15.7)	Smothery n = 23 (0.2)	Other namely…^3^: n = 547 (5.8)				Peanut n = 363 (3.8)	Nuts n = 33 (0.3)			>1 week n = 285 (3.0)
	n = 1 118 (1.4)												
	*[n = 262 (23*.*4)]*												
	Shellfish	Carrots (peel) n = 101 (0.1) *[n = 71 (70*.*3)]*	Itchy skin^d^ n = 1223 (12.9)	Increase saliva/mucus^e^ n = 22 (0.2)	Internist n = 125 (1.3)				Fish n = 209 (2.2)	Drupes n = 27 (0.3)			
	n = 918 (1.2)												
	*[n = 462 (50*.*3)]*												
	Peanut	Drupes n = 88 (0.1) *[n = 66 (75*.*0)]*	Vomiting n = 960 (10.1)	Swelling hands/feet n = 19 (0.2)	Gastroenterologist n = 114 (1.2)				Egg n = 112 (1.2)	Fruit n = 27 (0.3)			
	n = 904 (1.1)												
	*[n = 403 (44*.*6)]*												
	Almond	Nectarine n = 82 (0.1) *[n = 62 (75*.*6)]*	Itchy or teary eyes n = 870 (9.2)	Red bumps (hives) n = 17 (0.2)	Otolaryngologist n = 53 (0.6)				Almond n = 109 (1.1)	Banana n = 25 (0.3)			
	n = 781 (1.0)												
	*[n = 487 (62*.*4)]*												
	Cashew	Nuts/chocolate with- n = 39 (0.0) *[n = 22 (56*.*4)]*	Shortness of breath n = 626 (6.6)	Edema/ generalized swelling n = 12 (0.1)	‘‘Someone in the hospital” n = 52 (0.5)				Cashew n = 91 (1.0)	Nectarine n = 25 (0.3)			
	n = 556 (0.7)												
	*[n = 294 (52*.*9)]*												
	Pistachio	Brazil nuts n = 29 (0.0) *[n = 19 (65*.*5)]*	Redness of skin^d^ n = 741 (7.8)	Itchy palate n = 11 (0.1)	Pulmonologist n = 36 (0.4)				Soy n = 88 (0.9)	Dairy products n = 19 (0.2)			
	n = 390 (0.5)												
	*[n = 231 (59*.*2)]*												
	Fish	Pine nuts n = 26 (0.0) *[n = 11 (42*.*3)]*	Nasal symptoms n = 544 (5.3)	Strange feeling in mouth n = 9 (0.1)	Rheumatologist n = 5 (0.1)				Pistachio n = 24 (0.3)	Some nuts n = 17 (0.2)			
	n = 351 (0.4)												
	*[n = 185 (52*.*7)]*												
	Soy (milk)	Some nuts n = 24 (0.0) *[n = 13 (54*.*2)]*	Increase of AD^d^ n = 498 (4.7)	Swallowing problems n = 8 (0.1)	Neurologist n = 5 (0.1)				Sesam n = 23 (0.2)	Pine nuts n = 17 (0.2)			
	n = 318 (0.4)												
	*[n = 139 (43*.*7)]*												
	Egg n = 289 (0.4) *[n = 112 (38*.*8)]*	Dairy products n = 23 (0.0) *[n = 4 (17*.*4)]*	Coughing n = 444 (4.7)	Change of voice n = 8 (0.1)	Emergency physician n = 4 (0.0)				*Missing n = 276 (2*.*9)*	Celery n = 14 (0.1)			
	Sesame see n = 119 (0.2) *[n = 70 (58*.*8)]*	Celery n = 23 (0.0) *[n = 15 (65*.*2)]*	Urticaria^d^ n = 442 (4.7)	Tongue/mouth n = 7 (0.1)	Surgeon n = 4 (0.0)					Macadamia nuts n = 9 (0.1)			
		Pecan nuts n = 13 (0.0) *[n = 10 (76*.*9)]*	Palpitations n = 400 (4.2)	Swollen ears n = 4 (0.0)	Psychiatrist n = 3 (0.0)					Mixed nuts n = 6 (0.1)			
		Corn n = 12 (0.0) *[n = 1 (8*.*3)]*	Wheezing n = 301 (3.2)	Rash on face, neck, chest n = 3 (0.0)	Cardiologist n = 2 (0.0)					Corn n = 6 (0.1)			
		Mixed nuts n = 12 (0.0) *[n = 8*, *(61*.*5)]*	Dizziness n = 272 (2.9)	Metallic taste n = 3 (0.0)	Anesthetist n = 2 (0.0)					Pecan nuts n = 3 (0.0)			
		Macadamia nuts n = 12 (0.0) *[n = 9 (75*.*0)]*	Loss of consciousness n = 93 (1.0)	Anaphylactic shock n = 2 (0.0)	Ambulance staff n = 2 (0.0)					Brazil nuts n = 1 (0.0)			
		For less likely foods, see S1 Table.	Itchy skin at one location n = 407 (4.3)	For less likely symptoms, see S2 Table.	Alternative practitioner ^f^ n = 1181 (11.2)								
			Redness of skin at one location n = 327 (3.4)										
			Increase of AD at one location n = 204 (2.2)										
			Urticaria at one location n = 92 (1.0)										

Missing variables and answers such as: ‘I don’t know’ are shown in *Italics*. AD = atopic dermatitis, N^T^ = number of subjects and percentage of total (n = 78, 890), N^L^ = number of subjects classified as likely to have food allergy (*LikelyFA*) and percentage of N^T^.

^a^ 53 persons of these 69 410 reported any foods in question 1. These persons were defined as *Indeterminate*.

^b^ 39 persons of these 1 118 later indicated in the “Other(s)namely …..” option of question 1 or 2 that they had celiac disease. These persons were defined as *Indeterminate*.

^c^ The participants’ wording has been paraphrased and translated to approach the intent of the original. Therefore, some answers are non-specific and multiple answers could be entered.

^d^ At least multiple locations or generalized

^e^ Without reporting cow’s milk allergy

^f^ Patients were only defined as *Indeterminate* if this diagnosis by a (non-medical) alternative practitioner was not accompanied by one of the clinicians indicated above. This was the case in 983 out of 1181 = 83.2%.

^g^ These subjects are more extensively described in S4 Table.

The classification of which foods, symptoms and other characteristics of FA are consistent with immediate allergic reactions to foods is described in the supporting information. Subjects were classified as *Indeterminate* if they could not be classified as *NoFA* or *LikelyFA*. More specifically, this group included subjects with s-rFA who reported:

■ only symptoms to
■ foods uncommon or unproven to be elicitors of immediate allergic reactions and/or
■ only symptoms other than those consistent with immediate allergic reactions to foods and/or
■ only symptoms and/or foods associated with other disorders (such as lactose intolerance) and/or
■ one or more other (diagnostic) characteristics which are not consistent with allergic reactions to food (listed in Table 1 and Fig 1).

We hypothesized that the factors above indicate a potential false-positive case and to maximize the specificity of the questionnaire, we chose to exclude these patients from the *LikelyFA* group.

### Sensitivity analyses

By excluding subjects who were diagnosed by only a (non-medical) alternative practitioner, some truly food allergic patients might be excluded from the *LikelyFA* group. We tested this in a sensitivity analysis where we did not take this variable into account for the classification of subjects. Furthermore, we performed a sensitivity analysis in subjects with any clinician diagnosed *LikelyFA* only.

Subjects with s-rFA who reported other disorders (irritable bowel syndrome, Crohn’s disease, ulcerative colitis, rheumatoid arthritis or candida) in the ‘Other namely….’ option of question 1 and/or 2 of the FAQ were classified as *Indeterminate* since it was possible that these non-allergic diagnoses were the cause of the reported symptoms following the consumption of foods. A sensitivity analyses was performed regarding the influence of these disorders on the associations with H-RQOL.

In an additional sensitivity analyses we tested whether excluding subjects with only apple allergy, as a proxy for oral allergy syndrome, changed the results.

### Risk factors

The following risk factors were tested for association with all subgroups; gender, age, doctor’s diagnosis of asthma, nasal allergy including hayfever and eczema. The following self-reported risk factors were tested for association with *LikelyFA* compared to *NoFA*: ethnicity, any breastfeeding, duration of breastfeeding, living environment before the age of 5 years and birth mode (caesarean versus vaginal delivery). The definitions of these risk factors are described in the supporting information.

### Mental disorders and H-RQOL

For all subgroups, associations were studied with H-RQOL and three self-reported mental disorders: burn-out, depression and eating disorder. H-RQOL was determined using the RAND-36 questionnaire, which is the Dutch version of the SF-36 [16]. We calculated the general mental and physical component score (MCS and PCS) by performing a Z-score transformation of the subscales of the RAND-36 using the mean and standard deviation from the Dutch general population [17,18].

### Statistical analysis

Analyses were performed using SPSS 22 (IBM, Chicago, USA). Because of 14 tested variables, a two-sided Bonferroni-adjusted threshold of (0.05/14 = ) 3.57*10^−3^ was used. Associations were tested by logistic regression analysis adjusted for age, gender, asthma, nasal allergy and eczema since these variables were considered as potential confounders.

## Results

### Prevalence

In total, 79 964 subjects completed the second screening and 78, 890 subjects completed at least question 1 of the FAQ and were included in this study. Approximately 4.0% and 8.1% were classified as *LikelyFA* and *Indeterminate*, respectively (see Fig 1). Taken together, the prevalence of s-rFA was 12.1%. Apple was the most prevalent reported allergenic food, followed by cow’s milk and hazelnut. The proportion of subjects with s-rFA classified as *Indeterminate* is highly variable between the reported foods, as 77.9% of subjects reporting cow’s milk allergy was classified as *Indeterminate*, compared to 43.6% and 40.1% for apple and hazelnut, respectively (see Table 1). Interestingly, the majority of the subjects reported that they diagnosed their food allergy themselves (n = 6620, 69.8%) and only 13.2% of these subjects (n = 873) had also been diagnosed with food allergy by a clinician (including dieticians).

### Risk factors for food allergy

**Fig 2:**
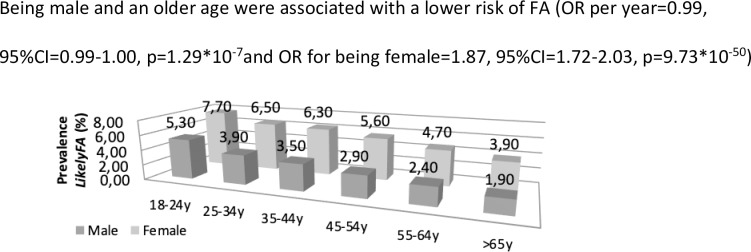
The prevalence of LikelyFA per age category and gender. Being male and an older age were associated with a lower risk of FA (OR per year = 0.99, 95%CI = 0.99–1.00, p = 1.29*10^-7^and OR for being female = 1.87, 95%CI = 1.72–2.03, p = 9.73*10^−50^).

As indicated in Table 2 and Fig 2, a younger age was associated with a higher risk of *LikelyFA* (OR = 0.99 per year, p = 1.29*10^−7^). In addition, females had a higher risk of *LikelyFA* compared to males (OR = 1.87, p = 9.73*10^−50^). Subjects classified as *LikelyFA* and *Indeterminate* more often reported asthma and eczema compared to subjects classified as *NoFA*. The prevalence of any nasal allergy including hay fever was double and triple that seen in subjects classified as *Indeterminate* and *LikelyFA*, respectively, compared to those classified as *NoFA* (46.0% and 64.9% compared to 22.8%, p<1.00*10^−250^ for both associations). Nasal allergy was the only atopic morbidity which was more prevalent in subjects classified as *LikelyFA* compared to those classified as *Indeterminate* after adjusting for potential confounders (OR = 2.10, p = 1.29*10^−56^).

**Table 2:** Characteristics of the study population.

Total n = 78,890	*NoFA* n = 69,357	*Indeterminate* n = 6,355	*Likely FA* n = 3,178	*LikelyFA vs NoFA*		*Indeterminate* vs *NoFA*		*LikelyFA* vs *Indeterminate*	
	n (valid%)	n (valid%)	n (valid%)	Unadjusted OR, 95%CI, p	Adjusted^a^ OR, 95%CI, p	Unadjusted OR, 95%CI, p	Adjusted^a^ OR, 95%CI, p	Unadjusted OR, 95%CI, p	Adjusted^a^ OR, 95%CI, p
**Male**	29,417 (42.4)	1,845 (29.0)	863 (27.2)	1.98	1.87	1.80	1.75	1.10	1.11
				1.83–2.14,	1.72–2.03	1.70–1.91,	1.65–1.86	1.00–1.21	1.00–1.22
				4.40*10^−63^	9.73*10^−50^	2.17*10^−93^	8.57*10^−81^	0.06	0.05
**Age in years** mean, SD	50.5, 12.6	49.5, 12.2	47.9, 12.2	0.98	0.99	0.99	1.00	0.99	0.99
				0.98–0.99	0.99–1.00	0.99–1.00	1.00–1.00	0.99–0.99	0.99–1.00
				2.39*10^−31^	1.29*10^−7^	5.17*10^−11^	0.61	2.41*10^−9^	0.03*10^−4^
**Asthma**	5,054 (7.4) m = 1 484	871 (13.7) m = 123	591 (18.6) m = 76	2.93	1.53	2.02	1.37	1.45	1.15
				2.66–3.21	1.39–1.70	1.87–2.18	1.27–1.49	1.29–1.63	1.02–1.29
				8.96*10^−111^	6.34*10^−17^	2.39*10^−71^	1.83*10^−14^	2.40*10^−10^	0.03
**Any form of nasal allergy including hay fever**	15,518 (22.8) m = 1 347	2,883 (46.0) m = 92	2,025 (64.9) m = 58	6.26	5.40	2.89	2.59	2.17	2.10
				5.80–6.75	4.99–5.85	2.74–3.04	2.46–2.74	1.98–2.37	1.92–2.30
				<1.00*10^−250^	<1.00*10^−250^	<1.00*10^−250^	<1.56*10^−250^	1.68*10^−65^	1.29*10^−56^
**Eczema**	9484 (13.9) m = 1 124	1550 (24.4) m = 74	887 (27.9) m = 51	2.45	1.68	2.03	1.64	1.21	1.04
				2.26–2.66	1.55–1.83	1.91–2.16	1.54–1.75	1.10–1.33	0.94–1.15
				3.14*10^−105^	5.46*10^−33^	2.69*10^−113^	1.63*10^−52^	1.21*10^−4^	0.49
**Burnout**	2,336 (3.5) m = 3 314	353 (5.7) m = 200	148 (4.5) m = 79	1.37	1.22	1.66	1.54	0.82	0.82
				1.15–1.62	1.02–1.46	1.48–1.86	1.37–1.74	0.68–1.00	0.67–1.01
				3.10*10^−4^	0.03	6.57*10^−18^	8.39*10^−13^	0.06	0.06
**Depression**	2,811 (4.2) m = 3 203	361 (5,9) m = 195	140 (4,8) m = 78	1.15	0.96	1.40	1.26	0.82	0.83
				0.97–1.36	0.81–1.15	1.25–1.57	1.18–1.48	0.67–0.99	0.68–1.01
				0.11	0.67	4.12*10^−9^	1.46*10^−4^	0.04	0.07
**Eating disorder**	384 (0.6) m = 3 364	64 (1.0) m = 206	26 (0.8) m = 82	1.45	1.01	1.80	1.34	0.81	0.80
				0.97–2.16	0.67–1.52	1.38–2.34	1.01–1.76	0.51–1.27	0.50–1.27
				0.07	0.97	0.16*10^−5^	0.04	0.35	0.34
**H-RQOL** median, IQR	m = 1 303	m = 91	m = 59	0.98	0.98	0.97	0.98	1.01	1.01
**PCS**	55.26,	53.94,	54.31,	0.97–0.98	0.98–0.99	0.97–0.98	0.97–0.98	1.00–1.01	1.00–1.01
	50.98–57.72	48.10–57.20	48.92–57.38	4.90*10^−26^	3.34*10^−11^	5.55*10^−72^	1.27*10^−43^	0.06	0.05
				0.98	0.99	0.98	0.98	1.00	1.01
**MCS**	53.37,	52.28,	52.58,	0.98–0.99	0.99–1.00	0.98–0.98	0.98–0.99	1.00–1.01	1.00–1.01
	49.02–56.36	46.72–55.82	47.02–55.87	1.62*10^−19^	0.52*10^−4^	7.27*10^−51^	8.26*10^−26^	0.16	0.03

Significant associations are highlighted. Vs = versus, CI = confidence interval, OR = odds ratio, H-RQOL = health-related quality of life, PCS = physical component score, MCS = mental component score, m = missing

^a^ adjusted for age in years, gender, asthma, nasal allergy, eczema (when not the tested variable). Relevant confounders changed the beta coefficient of the risk factor in the logistic regression by 10% or more [19] and are listed in S3 Table.

Approximately 76.2% of all subjects were breastfed and the prevalence of *LikelyFA* was 4.52% and 5.05% in the breastfed and not breastfed subjects, respectively. Breastfeeding was not associated with FA (OR = 1.04, 95%CI = 0.95–1.14, p = 0.37). In breastfed subjects, a shorter duration of breastfeeding was not associated with *LikelyFA* (OR = 0.98, p = 0.38, see S1 File of S1a Fig). Approximately 99.3% was of Western/Eastern European ethnicity. There was no association between FA and ethnicity (see S1 File of S1b Fig).

**Fig 3:**
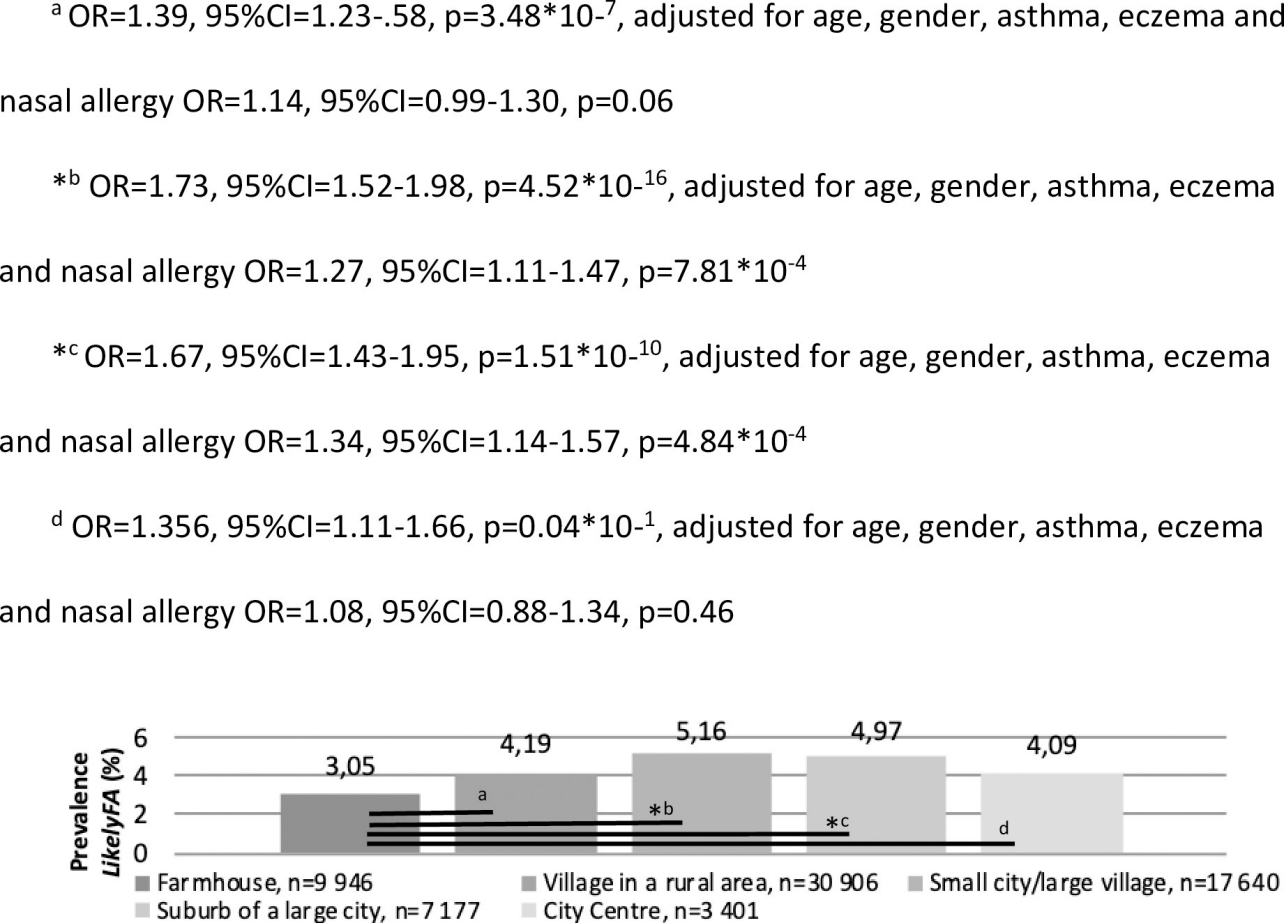
The prevalence of LikelyFA per answer as reported to the question ‘What is the best description of the place where you lived most of the time when you were younger than 5 years old?’. ^a^ OR = 1.39, 95%CI = 1.23-.58, p = 3.48*10−^7^, adjusted for age, gender, asthma, eczema and nasal allergy OR = 1.14, 95%CI = 0.99–1.30, p = 0.06. *^b^ OR = 1.73, 95%CI = 1.52–1.98, p = 4.52*10−^16^, adjusted for age, gender, asthma, eczema and nasal allergy OR = 1.27, 95%CI = 1.11–1.47, p = 7.81*10^−4^. *^c^ OR = 1.67, 95%CI = 1.43–1.95, p = 1.51*10−^10^, adjusted for age, gender, asthma, eczema and nasal allergy OR = 1.34, 95%CI = 1.14–1.57, p = 4.84*10^−4^. ^d^ OR = 1.356, 95%CI = 1.11–1.66, p = 0.04*10−^1^, adjusted for age, gender, asthma, eczema and nasal allergy OR = 1.08, 95%CI = 0.88–1.34, p = 0.46.

The prevalence of FA was 3.05% among adults who lived on a farm during childhood, which was lower compared to adults who lived in a small city/large village or a suburb of a large city (5.16% with OR = 1.27, p = 7.81*10^−4^ and 4.97%, OR = 1.34, p = 4.84*10^−4^, respectively, both adjusted for age, gender, asthma, eczema and nasal allergy, see Fig 3). Of the 1 818 subjects born by caesarean section, 4.73% (n = 86) was classified as *LikelyFA* which was not different among subjects born by vaginal delivery (4.61%, n = 2 933/63 640, OR = 0.97, 95%CI = 0.77–1.22, p = 0.79).

### Mental disorders and H-RQOL

There was no difference in the prevalence of reported burn-out, depression and eating disorder for the subjects classified as *LikelyFA* compared to those classified as *Indeterminate* or *NoFa* after adjustment for potential confounders, see Table 2. Interestingly, there were more subjects reporting burn-out and depression in the *Indeterminate* group, compared to the *NoFa* group (OR = 1.54, p = 8.39*10^-13^and OR = 1.26, p = 1.46*10^−4^, respectively). Both subjects classified as *LikelyFA* or *Indeterminate* scored lower compared to those classified as *NoFA* on the PCS and MCS measuring H-RQOL (see Table 2). There was no significant difference between subjects classified as *LikelyFA* and *Indeterminate* after Bonferroni correction for multiple testing (PCS: OR = 1.01, p = 0.05 and MCS: OR = 1.01, p = 0.03).

### Sensitivity analysis (see supporting information)

Subjects diagnosed by an alternative practitioner reported more foods, symptoms and characteristics inconsistent with FA compared to the remaining subjects with s-rFA. The prevalence of “any clinician diagnosed *LikelyFA*” (including dieticians) is only 38.5% of the prevalence of *LikelyFA*.

Excluding cases from the *Indeterminate* group with other disorders (e.g. celiac disease, lactose intolerance, ulcerative colitis) did not change the reported association with H-RQOL. Only 43.0% of the 430 subjects with only apple allergy were classified as *LikelyFA*. Excluding these cases did not change the results of any reported association.

## Discussion

Of this cohort of 78, 890 Dutch adults, approximately four percent reported FA with a culprit food, symptom and characteristics which we classified as consistent with *LikelyFA*. Additionally, eight percent was classified as having questionable, self-perceived FA without these features. Taken together, the prevalence of s-rFA was 12 percent. Interestingly, the majority of the subects with self-reported food allergy did not visit a clinician to confirm their self-made diagnosis.

The reported prevalence of food allergy is comparable to the in 1994 reported prevalence of s-rFA or food intolerance among 1483 Dutch adults, which was 12.4% [6] and the in 2015 reported prevalence of self-reported adverse reactions to Europrevall priority foods among 3 864 Dutch adults, which was 10.8% [8]. Although this would suggest that there has been no increase in the prevalence of food allergy over the last 24 years, we cannot exclude such an increase since our study population is larger and older compared to the study of 1994. Our study population with s-rFA had a median age of 45–54 years compared to 35–44 years in the study of 1994. Thus, age differences may have obscured an increase in prevalence since 1994.

The prevalence of s-rFA is higher than the prevalence of s-rFA in European adults in a meta analyses of 6 studies (5.1%). However, these studies were published between 2001 and 2008 and only one investigated western European subjects. Our prevalence of *LikelyFA*, 4.1%, is almost twice the prevalence of s-rFA plus sIgE positivity to at least one food in European adults, which was estimated at 2.2% [9].

Allergies associated with cross-reactivity to tree-pollen such as apple and hazelnut allergy were most commonly reported in subjects classified as *LikelyFA (*n = 1112 and n = 979, respectively) and 64.9% of subjects classified as *LikelyFA* reports any form of nasal allergy. Fortunately, progression to systemic symptoms and anaphylaxis is rare in these pollen-related allergies [20].

### Allergic comorbidities and food allergy

Both subjects classified as *LikelyFA* and *Indeterminate* reported asthma and eczema more often than subjects classified as *NoFa* and nasal allergy was the only allergic morbidity which was more prevalent in subjects classified as *LikelyFA* compared to those classified as *Indeterminate*. In addition, nasal allergy was a relevant confounder in the majority of the associations as indicated in S3 Table. This indicates that nasal allergy was more specifically associated with this questionnaire-based definition of *LikelyFA* which might be due to reports of foods cross-reacting with tree-pollen. Apple and hazelnut were the most often reported allergenic foods with a prevalence of 1.4% and 1.2%, respectively. Both food allergies can be caused by cross-reactivity and were documented to be foods to which adults are commonly sensitized (9.3% and 6.5%, respectively) [21,22].

### Other risk factors associated with food allergy

We report an association between the living environment during childhood and the risk of FA in adult life. Those who lived on a farm had a lower risk of FA compared to those who lived in a more urban environment. This confirms previous findings in 38 465 children of the US, in which there was an association between living in a rural area and having a lower risk of FA compared to living in an urban center [23]. In addition, our results indicate that this effect continues into adulthood. Several hypotheses have been put forward to explain this phenomenon, including the exposure to an increased microbial diversity, higher vitamin D levels and less exposure to ambient pollutants [23]. Environmental exposures may have epigenetic effects. Recently, DNA methylation differences in several genes including *STAT6* were reported in farmers’ compared to non-farmers’ children [24]. *STAT6* gene variants have previously been associated with DBPCFC diagnosed FA [25]. Exposure to farm milk was reported to be inversely associated with sensitization to foods in 7 606 children [26] and with higher numbers of regulatory T cells in 298 children [27]. This might be due to the consumption of bovine miRNAs in cow’s milk which are altered by high-heat treatment as applied to commercial milk [28].

We found a higher prevalence of FA in females and an older age was associated with a lower risk of FA. This replicates findings of a study of 2.7 million health records in the US which additionally reported a higher prevalence in subjects of Asian ethnicity [29]. We did not replicate this last finding since the association did not remain significant after adjusting for potential confounders, which was not performed previously. Moreover, our study was potentially underpowered to find an association with ethnicity since 99% of our population was of Western/Eastern European ethnicity. We cannot distinguish whether the association with age is based on a cohort effect or reflects a true association of FA with a younger age. The association of FA with gender was recently reviewed but the exact mechanism remains unknown [30]. The authors report lower IgG4 concentrations in females and discuss social and environmental gender-specific differences influencing allergen exposure [30,31]. Furthermore, estrogen enhances humoral immunity and sex hormone receptors have been found on the surface of lymphocytes and mast cells [30,32].

We were not able to replicate previous findings regarding the association between a longer duration of breastfeeding and a lower risk of FA [33]. This might be due to the studied population since this previous study used DBPCFC in children to distinguish food allergic cases from controls. Furthermore, the full range of duration of breastfeeding in months was studied instead of the ordinal answer options provided in the Lifelines questionnaire. In addition, we currently had no data available regarding family history of atopy, which is likely to influence this association through reverse causation [33].

### Characteristics of subjects classified as indeterminate

Approximately 66.7% of subjects with s-rFA was classified as *Indeterminate*. This is intermediate compared to the reported prevalences of unconvincing FA in studies from the USA and Central Brazil, which reported 19.4 and 85.6%, respectively [11,12]. The first study considered symptoms and timing of their onset. We used all criteria of the second study except the reproducibility and exclusion of the food from the daily diet which might explain our lower percentage of unconvincing FA. Interestingly, of subjects reporting cow’s milk allergy, 77.9% was classified as *Indeterminate*, compared to 43.6% and 40.1% for subjects reporting apple and hazelnut, respectively. This phenomenon was previously reported and might be due to confusion with lactose intolerance [12].

We defined subjects with a diagnosis by only an alternative practitioner as *Indeterminate* and showed that these subjects report more culprit foods and fewer symptoms, foods and characteristics that are consistent with FA when compared to other subjects with s-rFA. This suggests that alternative practitioners make more false positive diagnoses of FA than other caregivers. A review on this subject reported that there is no evidence for the diagnostic value of kinesiology and electrodermal testing, techniques frequently used by practitioners of alternative or complementary medicine [34].

We showed that subjects classified as *Indeterminate* reported more depression and burn-out than subjects classified as *NoFa*. This was not seen for subjects classified as *LikelyFA*. However, both subjects classified as *Indeterminate* and *LikelyFA* scored lower on both the MCS and PCS measuring H-RQOL compared to subjects classified as *NoFA*. Whether the poorer H-RQOL is a cause or consequence of questionable, self-perceived FA remains to be determined. Furthermore, this poorer H-RQOL could be caused by other comorbidities in these subjects, although the sensitivity analyses shows that this is not the case for celiac disease, irritable bowel syndrome, ulcerative colitis or lactose intolerance. The difference of the mean between subjects classified as *LikelyFA* and *NoFA* was 1.3 and 1.1 for the PCS and MCS, respectively. The standard deviation was 7.3 and 8,3 for the PCS and MCS, respectively, and a difference above half the standard deviation was previously suggested as the threshold for a clinically important difference [35]. Therefore, the clinical relevance of this reported difference in H-RQOL might be limited for individual subjects.

A recent review on psychosocial functioning of food allergic children and adolescents reported an association with internalizing of problems and bullying [36], which might influence the prevalence of mental disorders in adulthood. In addition, it was shown that atopic diseases and depression tend to occur together in families and twins and a small shared genetic effect for atopic diseases and depression was found [37,38]. Our data confirms that questionable, self-perceived FA is prevalent and associated with considerable psychological morbidity in adults. Thus, although the diagnosis may be questionable from a purely medical perspective, this is a group with a high morbidity that warrants more attention in future research. Interestingly, previous research indicated that in adults with suspected FA, a negative DBPCFC (FA ruled out) was associated with an improved H-RQOL 6 months later, especially when the test excluded all food allergies [39]. However, seemingly paradoxically, children also benefit from a positive DBPCFC confirming FA [39]. This suggests that subjects suffer from uncertainty regarding their food allergic status and this may partly explain the poorer H-RQOL in subjects in the indeterminate group.

### Strengths and limitations

This study is the largest population-based study on the prevalence of questionnaire-reported likely FA in Europe to date. As with most questionnaires on FA, it is not validated but based on expert opinion and previous studies defining FA by questionnaires [40,41]. In this classification, specificity was preferred over sensitivity to minimize the number of false positive cases within the *LikelyFA* group. Therefore, it is possible that there are subjects with FA in the *Indeterminate* group since there are probably subjects with FA who experience (less classic) allergic symptoms after eating (less classic) foods. Especially those who have experienced allergic reactions infrequently, or are allergic to a food rarely eaten or rarely involved in allergic reactions, will be prone to inappropriate exclusion from the *LikelyFA* group. The classification of reported foods was based on an extensive literature search but for some foods, literature was limited and their involvement in IgE-mediated FA cannot be excluded altogether. Within the Lifelines cohort there was no data available regarding sensitization or skin-prick testing, so we were unable to analyze the contribution of these parameters to the diagnosis. Validation of FA status of both subjects classified as *LikelyFA* and *Indeterminat*e by DBPCFC testing would be preferable but was not feasible.

## Conclusions

The prevalence of s-rFA among Dutch adults is considerable and only one-third reports characteristics consistent with FA. We found no evidence of an increased prevalence of s-rFA in Dutch adults compared to 1994. Females had a higher risk of FA and an older age was associated with a lower risk of FA. Living on a farm during childhood was associated with a lower risk during adulthood. Subjects with questionable, self-perceived FA reported a poorer H-RQOL compared to controls, just like subjects with likely FA. However, subjects classified as *Indeterminate* reported burn-out and depression more often compared to controls without FA. Whether this is a cause or consequence of perceived FA remains unclear. The high prevalence and considerable psychological burden of questionable, self-perceived FA makes this phenomenon a priority for future research. Furthermore, subjects with self-diagnosed food allergy should be stimulated to visit a clinician to confirm or reject this diagnosis which might improve their quality of life.

## Supporting information

S1 Table Frequency and classification of foods as reported in response to question 1: ‘*Which of the following food-items cause an allergic reaction*?*’ → ‘Other namely …*’. Subjects reporting foods from panel A were classified as likely food allergic when they met the criteria for the other questions as well. The foods in panel B may be reported by subjects along with foods from panel A, but are insufficient to classify a subject as ‘Likely having food allergy’ when reported alone. DBPCFC(s) = double-blind, placebo-controlled food challenge(s).The subjects’ wording has been paraphrased and translated to approach the intent of the original. Therefore, some answers are non-specific. ^#^ Common, less common, uncommon or unproven food allergy due to cross-reactive IgE sensitization to foods [1], see paragraph ‘*Classification of foods’*.
(DOCX)

S2 Table Frequency and classification of symptoms as reported in response to question 2: ‘*Which symptoms occur after eating or drinking the food item you are allergic to*?*’ → ‘Other namely …’*. Subjects reporting symptoms from panel A were classified as likely food allergic when they met the criteria for the other questions as well. The symptoms in panel B may be reported by subjects along with symptoms from panel A, but are insufficient to classify a subject as ‘Likely having food allergy’ when reported alone. The subjects’ wording has been paraphrased and translated to approach the intent of the original statement. CMA = cow’s milk allergy.
(DOCX)

S3 Table Relevant confounders which change the beta coefficient of the risk factor in the logistic regression analysis described in Table 2 by 10% or more [29]. Significant associations are highlighted. Vs = versus, H-RQOL = health-related quality of life, PCS = physical component score, MCS = mental component score, Y = age in years, G = gender, A = asthma, N = any form of nasal allergy including hay fever, E = eczema
(DOCX)

S4 Table Characteristics of the 2.142 subjects who developed immediate reactions to foods (within seconds). Of these subjects, the majority was classified as LikelyFA (n = 1301, 60.7%) and these cases are likely to represent severe cases of food allergy.
(DOCX)

S1 File (DOCX)

## Abbreviations

CI: confidence interval
FA: food allergy
FAQ: food allergy questionnaire
H-RQOL: health-related quality of life
LikelyFA: likely food allergy
MCS: mental component score
NoFA: no food allergy
PCS: physical component score
s-rFA: self-reported food allergy
OR: odds ratio
