# Supplementary material for: Likely questionnaire-diagnosed food allergy in 78, 890 adults from the northern Netherlands
Source: PLoS One. 2020 May 13;15(5):e0231818. doi: 10.1371/journal.pone.0231818 (PMC7219708; doi:10.1371/journal.pone.0231818)
Supplement: S1 Table — Subjects reporting foods from panel A were classified as likely food allergic when they met the criteria for the other questions as well. The foods in panel B may be reported by subjects along with foods from panel A, but are insufficient to classify a subject as ‘Likely having food allergy’ when reported alone. DBPCFC(s) = double-blind, placebo-controlled food challenge(s).The subjects’ wording has been paraphrased and translated to approach the intent of the original. Therefore, some answers are non-specific. # Common, less common, uncommon or unproven food allergy due to cross-reactive IgE sensitization to foods [1], see paragraph ‘Classification of foods’. (DOCX) [file pone.0231818.s001.docx]

**S1 Table. Frequency and classification of foods as reported in response to question 1: ‘*Which of the following food-items cause an allergic reaction?’ 🡪 ‘Other namely …*’.**

Subjects reporting foods from panel A were classified as likely food allergic when they met the criteria for the other questions as well. The foods in panel B may be reported by subjects along with foods from panel A, but are insufficient to classify a subject as ‘Likely having food allergy’ when reported alone.

DBPCFC(s) = double-blind, placebo-controlled food challenge(s).

The subjects’ wording has been paraphrased and translated to approach the intent of the original. Therefore, some answers are non-specific. ^#^ Common, less common, uncommon or unproven food allergy due to cross-reactive IgE sensitization to foods[1], see paragraph ’*Classification of foods’.*

| **Food** | **n** | **Important food allergen**[2] | **Europrevall priority food**[3] | **EAACI**  **classification**[1]  **#** | **Notes / references** |
| --- | --- | --- | --- | --- | --- |
| **A: Foods reported in the literature as commonly involved in immediate allergic reactions to food.** | | | | | |
| Kiwi | 783 | Yes | Yes | Common |  |
| Strawberry | 238 |  |  |  | [5] |
| Cherry | 169 | Yes |  | Common |  |
| Pear | 161 | Yes |  | Common |  |
| Peach | 159 | Yes | Yes | Common |  |
| Fruit | 141 |  |  |  | Not specific but suggestive for any fruit allergy. |
| Banana | 113 | Yes | Yes | Less common |  |
| Carrots (peel) | 101 | Yes | Yes | Common |  |
| Drupes/stone fruit | 88 | Yes |  |  | In Dutch this is a commonly used name for fruit containing a stone such as peach and cherry. |
| Nectarine | 82 |  |  | Common |  |
| Mixed nuts / Nuts / “Studentenhaver” / Chocolate with nuts | 51 |  |  |  | Not specific but suggestive for any nut allergy. |
| Brazil nuts | 29 |  |  |  | [6],[7] |
| Pine nuts | 26 |  |  |  | [8],[9] |
| Some nuts | 25 |  |  |  | Not specific but suggestive for any nut allergy. |
| Dairy products | 23 |  |  |  | Suggestive for cow’s milk allergy. |
| Celery | 22 | Yes | Yes | Common |  |
| Pecan nuts | 13 |  |  |  | [7] |
| Maize | 12 | Yes | Yes |  | [10],[11] |
| Macadamia nuts | 12 |  |  |  | [12] |
| **B: Less common allergenic foods and/or foods consistent with other disorders suspected in or by the subject** | | | | | |
| Pork  Meat  Beef (Red) | 264  19  12 |  |  | Uncommon |  |
| Chocolate | 224 |  |  |  | IgE-mediated chocolate allergy, as opposed to allergy to components of chocolate confections, including traces of other tree nuts, peanut, or cow’s milk is vanishingly rare and has not been reported in the modern literature in any study that used DBPCFC. [13] |
| Pineapple | 192 |  |  | Less common |  |
| Tomato | 177 |  | Yes | Less common |  |
| Bell pepper | 160 |  |  |  | Limited literature: only few cases were reported[14],[15]. |
| Sugar  Fructose | 133  11 |  |  |  | Intolerance to carbohydrates is the most common type of non-immune-mediated adverse food reactions.[16]  For fructose: non-allergic hereditary fructose aldolase B deficiency which is a rare deficiency of fructose carrier GLUT5 in the enterocytes in small intestines[2]. |
| MSG  Chinese food | 115  17 |  |  |  | Non-allergic adverse reactions to monosodium glutamate (MSG) begin 15-20 minutes after the meal and last for 2 hours.[2] In a DBPCFC study, objective reactions to MSG were observed in only 2 of 130 self-selected MSG-reactive adult volunteers[17].  Chinese food contains MSG but might as well contain cashew nuts and sesame seeds. However, non-allergic adverse reactions to MSG are commonly referred to as the Chinese restaurant syndrome. |
| Gluten | 104 |  |  |  | Celiac disease[2]. |
| Melon (all sorts) | 98 |  | Yes | Unproven |  |
| Onion | 95 |  |  | Less common |  |
| Herbs/spices  Curry(powder)  Cinnamon | 84  25  30 |  |  | Less common |  |
| Lactose | 83 |  |  |  | Lactase deficiency. |
| Potatoes (peel) | 82 | Yes |  | Common | Potato peel and raw potatoes are in the Netherlands generally not eaten and are therefore less likely to be involved in an IgE mediated allergic reaction to an ingested food. |
| Cheese (all different sorts) | 79 |  |  |  | Conflicting data about a non-allergic adverse reaction to tyramine in cheese[2],[18]. Furthermore, symptoms might also be due to a non-allergic lactase deficiency. |
| Citrus fruits  Orange | 70  71 |  |  |  | Not described in literature. Interestingly there is a study describing a protective effect of citrus on allergic responses[19]. |
| Cabbage | 66 |  |  | Less common |  |
| Mushrooms | 65 |  |  |  | Non-allergic adverse reaction to serotonin in mushrooms[2]. |
| ‘’Additives’’  Flavoring agents  Coloring agents  Artificial additives Sweeteners | 11  64  49  38  24 |  |  |  | Adverse reactions, including urticaria or angioedema, asthma, or anaphylaxis, to many additives have not been found in methodologically sound studies and must be extremely rare if they occur at all[13]. For sulphites, see wine. |
| Plums | 59 | Yes |  | Uncommon |  |
| Wine (red, white)  Histamine  Sulphite | 54  17  12 |  |  |  | Conflicting data about non-allergic adverse reaction to histamine in wine with flushing, headache and nausea[2],[18],[20] or sulphite induced asthma[2],[20] .  Reviews on this topic conclude that the literature is limited [18],[21]. There is only one conclusive study, showing no relation between histamine content of red wine and tolerance to wine in patients with a history of red-wine provoked asthma[18].  Sulphites are described to potentially cause urticaria, angio-oedema or less commonly anaphylaxis and rhinitis although the mechanism is unknown, summarized by Skypala et al[20]. |
| Pungent spices  Peppers | 54  32 |  |  | Less common |  |
| Coffee  Caffeine | 52  25 |  |  |  | Non-allergic adverse reaction to caffeine[2]. |
| Fat | 51 |  |  |  | Not described in literature. |
| Garlic | 50 |  |  | Less common |  |
| Spinach | 48 |  |  |  | Non-allergic adverse reaction to histamine in spinach with flushing, headache and nausea[2] |
| Yeast | 41 |  |  |  | Non-allergic adverse reaction to histamine in yeast[2]. |
| Avocado | 41 | Yes |  | Less common |  |
| Alcohol | 36 |  |  |  | Non-allergic adverse reaction due to a polymorphism of the aldehyde dehydrogenase gene ALDH resulting in deficiency of ALDH which metabolizes alcohol in the liver in asians[2]. |
| Lettuce/ arugula | 36 |  |  | Uncommon |  |
| ‘’Nightshade’’ | 27 |  |  |  | Includes tomato, bell pepper and potato. Two out of three are classified as less likely to be associated with food allergy. Therefore, the combination of all three would be less likely to be associated with food allergy. |
| Mango | 27 |  |  | Less common |  |
| Rye | 27 |  |  |  | Limited literature, only a case study about rye dependent exercise-induced anaphylaxis[22]. Furthermore the symptoms might also be caused by Celiac disease. |
| Grapes | 26 | Yes |  | Less common |  |
| Legumes | 23 |  |  |  | A limited number of positive DBPCFCs to specific legume foods is reported in literature[23] and legumes were reported to be involved in 4 cases of anaphylaxis in Italian adults[15]. However, cross-reactivity to legumes without soy or peanut allergy is uncommon and avoidance of all legumes is generally unwarranted[13]. Of these 23 subjects, 5 reported peanut, and 4 reported soy allergy. |
| Apricots | 23 | Yes |  | Uncommon |  |
| Honey | 22 |  |  |  | Is reported to give symptoms in patients suffering from Irritable Bowel Syndrome[21]. There is only limited literature regarding honey allergy but it is reported to be rare[24],[25]. |
| Beer | 18 |  |  |  | Limited literature: In several cases, lipid transfer proteins have been identified as allergens in cases of reactions to beer[26]. |
| Ice cream | 17 |  |  |  | Not described in literature. |
| Cacao | 16 |  |  |  | Limited literature: cacao is a tree nut and some sequence homology has been found between cacao vicilin seed storage protein and walnut vicilin. However, cacao seeds undergo extensive processing, with the end result being that in commercial chocolate the proteins exist in an insoluble, complex form[13]. |
| Figs | 16 | Yes |  | Uncommon |  |
| Rice | 11 |  |  |  | Rarely, allergic reactions to rice can arise in patients with peach allergies[27]. |
| **372 subjects only reported an allergenic food, other than those described above**  **312 subjects did not specify an allergenic food, stating other information such as ‘ I don’t know’, ‘currently under investigation’ or ‘not applicable’.** | | | | | |
