# Supplementary material for: Likely questionnaire-diagnosed food allergy in 78, 890 adults from the northern Netherlands
Source: PLoS One. 2020 May 13;15(5):e0231818. doi: 10.1371/journal.pone.0231818 (PMC7219708; doi:10.1371/journal.pone.0231818)
Supplement: S2 Table — Subjects reporting symptoms from panel A were classified as likely food allergic when they met the criteria for the other questions as well. The symptoms in panel B may be reported by subjects along with symptoms from panel A, but are insufficient to classify a subject as ‘Likely having food allergy’ when reported alone. The subjects’ wording has been paraphrased and translated to approach the intent of the original statement. CMA = cow’s milk allergy. (DOCX) [file pone.0231818.s002.docx]

**S2 Table. Frequency and classification of symptoms as reported in response to question 2: ‘*Which symptoms occur after eating or drinking the food item you are allergic to?’ 🡪 ‘Other namely …’.***

Subjects reporting symptoms from **panel A** were classified as likely food allergic when they met the criteria for the other questions as well. The symptoms in **panel B** may be reported by subjects along with symptoms from panel A, but are insufficient to classify a subject as ‘Likely having food allergy’ when reported alone.

The subjects’ wording has been paraphrased and translated to approach the intent of the original statement. CMA= cow’s milk allergy.

| **Symptom** | **n =2126** | **Notes** |
| --- | --- | --- |
| **A: Symptoms consistent with immediate allergic reactions to foods** | | |
| Painful mouth/tongue/”blisters” in mouth | 157 |  |
| Increase saliva/mucus | 22 | Without reporting CMA or dairy products allergy. |
| Red/Swollen eyes | 47 |  |
| Sneezing | 47 |  |
| Strange feeling or painful throat | 40 |  |
| Swollen face/ Quincke’s edema | 27 |  |
| Swollen throat | 24 |  |
| Smothery | 23 |  |
| Swelling hands/feet | 19 |  |
| Red bumps (hives) | 17 |  |
| Itchy palate | 11 |  |
| Edema/ generalized swelling | 9 |  |
| Strange feeling in mouth | 9 |  |
| Problems swallowing | 8 |  |
| Change of voice | 8 |  |
| Swelling of tongue/mouth | 7 |  |
| Swollen ears | 4 |  |
| Rash on face, chest and neck | 3 |  |
| Metallic taste in mouth | 3 |  |
| Anaphylactic shock | 2 |  |
| **B: Symptoms other than those consistent with immediate allergic reactions to foods*** | | |
| Headache/migraine | 218 |  |
| Puffy feeling | 189 |  |
| Tired/drowsy | 140 |  |
| Stomach ache/ heartburn | 119 | Gastro Esophageal Reflux disease. |
| Painful abdomen/intestines | 118 |  |
| Pimples/acne | 100 |  |
| Flatulence | 90 |  |
| Constipation | 60 |  |
| Mouth ulcers | 47 |  |
| Increased saliva/mucus | 42 | As sole symptom with “CMA or dairy products allergy”. |
| Painful joints | 38 |  |
| Not feeling well/depressed/bad mood | 36 |  |
| Change in stool (frequency/composition) | 34 | Excluding diarrhea. |
| Sweating | 33 |  |
| Feeling restless | 31 |  |
| Painful muscles | 28 |  |
| Strange taste in mouth | 22 | Excluding metallic taste. |
| Chest pain | 23 |  |
| Fluid retention | 20 |  |
| Increase in temperature/fever | 20 |  |
| Painful esophagus | 17 | Eosinophilic esophagitis. |
| Burping | 16 |  |
| Swollen gland(s) | 11 |  |
| Cold sore | 8 |  |
| Infected sinus | 7 |  |
