# Supplementary material for: Likely questionnaire-diagnosed food allergy in 78, 890 adults from the northern Netherlands
Source: PLoS One. 2020 May 13;15(5):e0231818. doi: 10.1371/journal.pone.0231818 (PMC7219708; doi:10.1371/journal.pone.0231818)
Supplement: S3 Table — Significant associations are highlighted. Vs = versus, H-RQOL = health-related quality of life, PCS = physical component score, MCS = mental component score, Y = age in years, G = gender, A = asthma, N = any form of nasal allergy including hay fever, E = eczema (DOCX) [file pone.0231818.s003.docx]

**S3 Table. Relevant confounders which change the beta coefficient of the risk factor in the logistic regression analysis described in Table 2 by 10% or more[28].**

|  | ***LikelyFA vs NoFA*** | ***Indeterminate* vs *NoFA*** | ***LikelyFA* vs *Indeterminate*** |
| --- | --- | --- | --- |
| **Male** | - | - | N |
| **Age in years** | A, N, E | G, A, N, E | N |
| **Asthma** | N, E | N, E | N |
| **Any form of nasal allergy including hay fever** | - | - | - |
| **Eczema** | N | N | Y, A, N |
| **Burnout** | N | - | - |
| **Depression** | G, A, N, E | G | N |
| **Eating disorder** | Y, G, A, N, E | G, E | A, E |
| **H-RQOL**  **PCS**  **MCS** | Y, A, N  Y, G, N, E | A  G | Y, A  Y |

Significant associations are highlighted**.** Vs = versus, H-RQOL = health-related quality of life, PCS= physical component score, MCS= mental component score, Y= age in years, G=gender, A=asthma, N=any form of nasal allergy including hay fever, E=eczema.
