# Supplementary material for: Likely questionnaire-diagnosed food allergy in 78, 890 adults from the northern Netherlands
Source: PLoS One. 2020 May 13;15(5):e0231818. doi: 10.1371/journal.pone.0231818 (PMC7219708; doi:10.1371/journal.pone.0231818)
Supplement: S4 Table — Of these subjects, the majority was classified as LikelyFA (n = 1301, 60.7%) and these cases are likely to represent severe cases of food allergy. (DOCX) [file pone.0231818.s004.docx]

**S4 Table. Characteristics of the 2.142 subjects who developed immediate reactions to foods (within seconds). Of these subjects, the majority was classified as LikelyFA (n=1301, 60.7%) and these cases are likely to represent severe cases of food allergy.**

|  | ***Indeterminate (n=841)***  **n(valid %)** | ***LikelyFA (n=1301)***  **n(valid %)** |
| --- | --- | --- |
| **Male;** | 236 (28.1) | 328 (25.2) |
| **Age in years; mean, SD** | 46.8, 11.5 | 46.1, 11.9 |
| **Top five most common reported foods** | Apple (300, 35.7)  Hazelnut (198, 23.5)  Walnut (168, 20.0)  Kiwi (109, 13.0)  Cow’s milk (87, 10.3) | Apple (650, 50.0)  Hazelnut (556, 42.7)  Walnut (478, 36.7)  Kiwi (295, 22.7)  Almond (282, 21.7) |
| **Asthma** | 166 (20.2) | 275 (21.5) |
| **Any form of nasal allergy including hay fever** | 541 (65.1) | 988 (76.9) |
| **Eczema** | 219 (26.3) | 382 (29.7) |
