# Supplementary material for: Likely questionnaire-diagnosed food allergy in 78, 890 adults from the northern Netherlands
Source: PLoS One. 2020 May 13;15(5):e0231818. doi: 10.1371/journal.pone.0231818 (PMC7219708; doi:10.1371/journal.pone.0231818)
Supplement: S1 File — (DOCX) [file pone.0231818.s005.docx]

Supporting information of

**Methods**

**Classification of foods**

Following question 1, subjects could select food items from a list (Table 1). These foods are all generally regarded as common allergenic foods[1]–[3]. Subjects could also report (additional) foods by use of the option ‘Other namely … ‘. If the subjects’ wording was unclear or non-specific (such as complex food products or dishes), the answers given were discussed with an experienced allergist. Foods that were reported in the option ‘*Other namely’* by more than 1:1 000 subjects with self-reported food allergy (s-rFA) were considered as being a food eliciting immediate allergic reactions for our study population if such a food was described as being a known allergenic food in either the EAACI position paper[1] or Middleton’s Allergy Principles and Practice[2] or if it was listed as an Europrevall priority food[1],[3]. For foods which were not mentioned in these overviews a PUBMED search was conducted using the reported food and food allergy as MESH terms. Subjects reporting only foods for which a non-allergic adverse reaction was possible, foods described by the EAACI as uncommon or unproven elicitors of IgE mediated reactions to foods^[^1^]^ or foods which were not unambiguously reported as elicitors of immediate allergic reactions were classified as *Indeterminate*. Answers which were reported in the ‘Other namely…’ option by less than 1:1 000 subjects were screened by the authors for any potential common allergenic foods and these were then reviewed as described above. For the list of foods reported by subjects and the relevant literature see Table 1 and S1 Table.

**Classification of symptoms**

Subjects responding to the second question (‘Which symptom occurs after eating or drinking the food item that you are allergic to?’) could select symptoms from a predefined list or enter a symptom by use of the option ‘Other namely …’. All symptoms were classified as being consistent or inconsistent with immediate allergic reactions to foods by the authors based on clinical experience and expert opinion, see Table 1. Symptoms reported by individuals which did not appear on the predefined list were assessed by the authors and classified as above. These were summarized in S2 Table.

**Classification of other characteristics of food allergy**

The following other characteristics of food allergy were used to further classify the subjects. As shown in Table 1, question 6a-d focused on the symptoms occurring after eating the food item that triggered the most severe reaction. Following question 6b ‘*How quickly do these symptoms appear?*’, subjects could choose different options, such as ‘*immediately (seconds)*’ or ‘*after a few hours’*. Subjects entering the option ‘*after a day or more*’ were classified as *Indeterminate*, since this interval is not consistent with immediate allergic reactions to foods. Following question 6c *‘Which amount causes these symptoms?’*, subjects answering *‘a normal portion or more’* were defined as *Indeterminate* since immediate allergic reactions to foods classically appear after smaller portions such as the other answer possibilities ‘*traces (invisible)*’ or ‘*crumbs - few bites/sips*’. The same applies for subjects answering *‘>1 week’* to question 6d *‘How long do these symptoms persist?’.* Classic food allergy symptoms tend to resolve within several days and symptoms lasting for over a week are more likely to be associated with some other diagnosis, such as non-allergic reactions, intolerances to food or unrelated conditions.

**Diagnosis and treatment**

In question 3, subjects were asked ‘*Who diagnosed the food allergy?*’. We did not interpret a diagnosis of an alternative practitioner as likely to be consistent with food allergy. Therefore, subjects were defined as *Indeterminate* if a diagnosis by a (non-medical) alternative practitioner was not accompanied by the diagnosis of a medical clinician. In question 4, patients were asked whether they had ‘*an adrenalin auto-injector /Epipen /Anapen/ Jext’*. This question was not used to classify subjects. Question 5a and b were regarding any 2-day double blind oral food challenges undergone by the subject and the outcome of this test. Subjects with s-rFA who reported such a test with a negative result were also classified as *Indeterminate* since these subjects persisted in considering themselves to be food allergic despite the negative test result.

**Risk factors**

Age was studied as a continues variable and visualized in the same categories as previously published[4], 18-24, 25-34, 35-44, 45-54, 55-64, 65+ years.

Asthma was defined based on a self-reported doctor’s diagnosis of asthma (‘Have you ever had asthma’ followed by ‘If you have ever had asthma, was this confirmed by a doctor?’) or 2 or more symptoms combined with the use of asthma medication. ‘Wheezing’, an ‘attack of shortness of breath during daytime while at rest’ or ‘waking up at night with shortness of breath‘ were considered as asthma symptoms. Eczema, burn-out, depression and eating disorder were all options following the question ‘Could you indicate which of the following disorders you have (had)?’ Subjects who did not answered for any of these disorders a ‘yes’ but completed this question by indicating another disorder, or answering “none”, were classified as not (ever) having eczema, burn-out, depression or eating disorder.

Subjects were asked ‘How long were you breastfed as a baby’ and could select 1) ‘I was not breastfed’, 2) ‘I was breastfed but I don’t know for how long’, 3) ‘0-2 weeks’, 4) ‘2-4 weeks’, 5) ‘1-3 months’, 6) ‘3-6 months’, 7) ‘6-12 months’, 8) ‘for more than one year’ or 9) ‘I don’t know whether I was breastfed’. For breastfeeding (any duration versus no breastfeeding), answers 2-8 were compared to 1. For the duration of breastfeeding in breastfed children, answers 3-8 were studied in which category 3 and 4 were taken together to improve distribution, since the numbers in both categories were low.

Ethnicity was studied by the provided answer options for the question: ‘Which of the following populations do you consider yourself belonging to?’, see S1b Fig. Living environment was studied by the answer options provided for the question: ‘What is the best description of the place where you lived most of the time when you were younger than 5 years old?’, see Fig 4. Mode of delivery was studied by the provided answer options for the question: ‘How were you born?’. Subjects could select 1) ‘Normal vaginal delivery’, 2) ‘Vaginal delivery with the use of forceps or vacuum pump’, 3) ‘Caesarean section’ and 4) ‘I do not know’. Answers 1 and 2 were compared to 3.

**Results**

**S1A and B Figs. The prevalence of LikelyFA per duration of breastfeeding (panel A) and population as reported by the question ‘Which of the following populations do you consider yourself belonging to?’ (panel B)*.***

**A**. There was no significant association between the duration of breastfeeding and food allergy (OR=0.96, p=0.07, adjusted for age, gender, asthma, nasal allergy and eczema: OR=0.98, 95%CI=0.93-1.03, p=0.38).

**B.** There was no significant association between ethnicity and food allergy after correction for confounding factors.

^a^ OR=1.69, p=0.05*10^-1^, adjusted for age, gender, asthma, nasal allergy and eczema OR=1.96, 95%CI=0.90-4.23, p=0.09.

^b^ OR=1.99, p=0.03*10^-1^, adjusted for age, gender, asthma, nasal allergy and eczema OR=1.42, 95%CI=0.90-2.27, p=0.14

**A**

**B**

^b^

^a^

**Sensitivity analyses**

Of all subjects with s-rFA, approximately 10.4% were only diagnosed by an alternative practitioner which we did not interpret as being consistent with likely food allergy. Had we done so, 232 out of the 983 subjects (23.6%) with s-rFA with a diagnosis by an alternative practitioner without a diagnosis by another clinician would be classified as *LikelyFA*, based on the other criteria. This is significantly less than the percentage classified as *LikelyFA* of the remaining subjects with s-rFA (3178 out of 8497 = 37.4%, p=1.40E-17). Subjects with a diagnosis by an alternative practitioner without a diagnosis by another clinician reported more culprit foods compared to the other subjects with s-rFA (median 2 vs median 1, Mann-Whitney U test p=0.00E-0).

Some subjects reported celiac disease (n=39), lactose intolerance (n=71), and other diagnoses (irritable bowel syndrome, Crohn’s disease, ulcerative colitis, rheumatoid arthritis or candida, n=36) in the “other namely …..” option of question 1 and 2 of the FAQ and were classified as *Indeterminate*. Excluding these cases from the *Indeterminate* group did not change the reported association with H-RQOL (data not shown).

There were 419 subjects who only reported apple allergy. Of these, 180 (43.0%) were classified as *LikelyFA.* When these 180 subjects were classified as *Indeterminate* (instead of *LikelyFA*), the prevalence of *LikelyFA* would be 3.8% (2 998/78 890) instead of 4.0%. Excluding these cases from the *LikelyFA* group did not change any reported associations with *LikelyFA* (data not shown).

Approximately 61.9% and 76.0% of subjects classified as *LikelyFA* and *Indeterminate*, respectively, diagnosed the (perceived) food allergy themselves. The prevalence of “any clinician diagnosed *LikelyFA*” (*acd-LikelyFA*, including dieticians) is 1.54% which is only 38.5% of the prevalence of *LikelyFA.* Most associations remained significant after subjects without any diagnosis of any clinician were excluded from the *LikelyFA* group. However, the associations regarding the living environment during infancy did not reach significance (after adjusting for multiple testing).

## **SReferences**

1. Werfel T, Asero R, Ballmer-Weber BK, Beyer K, Enrique E, Knulst AC, et al. Position paper of the EAACI: Food allergy due to immunological cross-reactions with common inhalant allergens. Allergy Eur J Allergy Clin Immunol. 2015;70(9):1079–90.

2. Nowak-Wegrzyn A, Burks AW, and Sampson HA. Reactions to foods. In: Adkinson NF, Bochner BS, Burks AW, Busse WW, Holgate ST, Lemanske RF, et al., editors. Middleton’s Allergy Principles and Practice. 8th ed. Philadelphia: Elsevier, Saunders; 2014. p. 1310–39.

3. Fernandez-Rivas M, Barreales L, Mackie AR, Fritsche P, Vazquez-Cortes S, Jedrzejczak-Czechowicz M, et al. The EuroPrevall outpatient clinic study on food allergy: Background and methodology. Allergy Eur J Allergy Clin Immunol. 2015;70(5):576–84.

4. Niestijl Jansen JJ, Kardinaal AFM, Huijbers G, Vlieg-Boerstra BJ, Martens BPM, and Ockhuizen T. Prevalence of food allergy and intolerance in the adult Dutch population. J Allergy Clin Immunol. 1994;93(2):446–56.

5. Hassan A, and Venkatesh Y. An overview of fruit allergy and the causative allergens. Eur Ann Allergy Clin Immunol. 2015;47(6):180–7.

6. Rayes H, Raza A A, Williams A, Matthews S, and Arshad SH. Specific IgE to recombinant protein (Ber e 1) for the diagnosis of Brazil nut allergy. Clin Exp Allergy. 2016;46(4):654–6.

7. Couch C, Franxman T, and Greenhawt M. Characteristics of tree nut challenges in tree nut allergic and tree nut sensitized individuals. Ann Allergy, Asthma Immunol. 2017;

8. Cabanillas B, Crespo JF, Maleki SJ, Rodriguez J, and Novak N. Pin p 1 is a major allergen in pine nut and the first food allergen described in the plant group of gymnosperms. Food Chem. 2016;210:70–7.

9. Cabanillas B, and Novak N. Allergic reactions to pine nut: A review. J Investig Allergol Clin Immunol. 2015;25(5):329–33.

10. Pastorello EA, Farioli L, Pravettoni V, Scibilia J, Conti A, Fortunato D, et al. Maize food allergy: Lipid-transfer proteins, endochitinases, and alpha-zein precursor are relevant maize allergens in double-blind placebo-controlled maize-challenge-positive patients. Anal Bioanal Chem. 2009;395(1):93–102.

11. Scibilia J, Pastorello EA, Zisa G, Ottolenghi A, Ballmer-Weber B, Pravettoni V, et al. Maize food allergy: A double-blind placebo-controlled study. Clin Exp Allergy. 2008;38(12):1943–9.

12. De Knop KJ, Hagendorens MM, Bridts CH, Stevens WJ, and Ebo DG. Macadamia nut allergy: 2 Case reports and a review of the literature. Acta Clin Belg. 2010;65(2):129–32.

13. Chapman JA, Bernstein IL, Lee RE, Oppenheimer J, Editors A, Nicklas RA, et al. Food allergy : a practice parameter. Ann Allergy Asthma Immunol. 2006;96(S1):1–68.

14. García-Menaya JM, Cordobés-Durán C, Bobadilla-González P, Ledesma A, Pérez-Rangel I, Sánchez-Vega S, et al. Anaphylactic reaction to bell pepper (Capsicum annuum) in a patient with a latex-fruit syndrome. Allergol Immunopathol (Madr). 2014;42(3):263–5.

15. Asero R, Antonicelli L, Arena A, Bommarito L, Caruso B, Colombo G, et al. Causes of food-induced anaphylaxis in italian adults: A multi-centre study. Int Arch Allergy Immunol. 2009;150(3):271–7.

16. Canani RB, Pezzella V, Amoroso A, Cozzolino T, Di Scala C, and Passariello A. Diagnosing and treating intolerance to carbohydrates in children. Nutrients. 2016;8(3).

17. Geha RS, Beiser A, Ren C, Patterson R, Greenberger PA, Grammer LC, et al. Multicenter, double-blind, placebo-controlled, multiple-challenge evaluation of reported reactions to monosodium glutamate. J Allergy Clin Immunol. 2000;106(5):973–80.

18. Jansen SC, van Dusseldorp M, Bottema KC, and Dubois AEJ. Intolerance to dietary biogenic amines: a review. Ann Allergy Asthma Immunol. 2003;91(3):233–40; quiz 241–2, 296.

19. Chung MY, Shin HS, Choi DW, and Shon DH. Citrus Tachibana Leaf Extract Mitigates Symptoms of Food Allergy by Inhibiting Th2-Associated Responses. J Food Sci. 2016;81(6):H1537–45.

20. Skypala IJ, Williams M, Reeves L, Meyer R, and Venter C. Sensitivity to food additives, vaso-active amines and salicylates: a review of the evidence. Clin Transl Allergy. 2015;5(1):34.

21. Skypala IJ, Venter C, Meyer R, deJong NW, Fox AT, Groetch M, et al. The development of a standardised diet history tool to support the diagnosis of food allergy. Clin Transl Allergy. 2015;5:7.

22. García-Menaya JM, Chiarella GM, Cordobés-Durán C, Mahecha AC, and Bobadilla-González P. Rye-dependent exercise-induced anaphylaxis. Ann Allergy, Asthma Immunol. 2016;117(5):566–8.

23. Martínez San Ireneo M, Ibáñez MD, Sánchez J-J, Carnés J, and Fernández-Caldas E. Clinical features of legume allergy in children from a Mediterranean area. Ann Allergy Asthma Immunol. 2008;101(2):179–84.

24. Aguiar R, Cabral Duarte F, Mendes A, Bartolome B, and Barbosa MP. Anaphylaxis caused by honey: a case report. Asia Pac Allergy. 2012;2(1):76–85.

25. Cifuentes L. Allergy to honeybee … not only stings. Curr Opin Allergy Clin Immunol. 2015;15(4):364–8.

26. Asero R, Mistrello G, Roncarolo D, and Amato S. Relationship between peach lipid transfer protein specific IgE levels and hypersensitivity to non-Rosaceae vegetable foods in patients allergic to lipid transfer protein. Ann Allergy Asthma Immunol. 2004;92(2):268–72.

27. Pastorello EA, Scibilia J, Farioli L, Primavesi L, Giuffrida MG, Mascheri A, et al. Rice allergy demonstrated by double-blind placebo-controlled food challenge in peach-allergic patients is related to lipid transfer protein reactivity. Int Arch Allergy Immunol. 2013;161(3):265–73.

28. Twisk JWR. Applied multilevel analysis: A practival guide. Cambridge university press; 2006.
